# A Lateral Line Specific Mucin Involved in Cupula Growth and Vibration Detection in Zebrafish

**DOI:** 10.3390/ijms26020708

**Published:** 2025-01-15

**Authors:** Ziyue Ma, Yixuan Tian, Yingying Wang, Chenghao Wang, Jian Wang, Chunxin Fan

**Affiliations:** 1 Institute for Marine Biosystem and Neuroscience, International Center for Marine Studies, Shanghai Ocean University, Shanghai 201306, China; m220100114@st.shou.edu.cn (Z.M.); 2211406@st.shou.edu.cn (Y.T.); m230100045@st.shou.edu.cn (Y.W.); chenghaow5@student.unimelb.edu.au (C.W.); 2 Key Laboratory of Exploration and Utilization of Aquatic Genetic Resources, Ministry of Education, Shanghai Ocean University, Shanghai 201306, China; 3 International Research Center for Marine Biosciences, Ministry of Science and Technology, Shanghai Ocean University, Shanghai 201306, China; 4 Marine Biomedical Science and Technology Innovation Platform of Lingang New Area, Shanghai 201306, China

**Keywords:** lateral line, zebrafish, Mucin-5AC, cupula, startle response

## Abstract

The lateral line system in fish is crucial for detecting water flow, which facilitates various behaviors such as prey detection, predator avoidance, and rheotaxis. The cupula, a gelatinous structure overlaying the hair cells in neuromasts, plays a key role in transmitting mechanical stimuli to hair cells. However, the molecular composition of the cupula matrix remains poorly understood. In this study, we found that Mucin-5AC, a novel family of mucin proteins, composed of 2–27 cysteine-rich domains, presents in cartilaginous and bony fishes. Using in situ hybridization and transgenic reporter assays, we demonstrated that zebrafish *muc5AC* is specifically expressed in the support cells of neuromasts. Knockdown of *muc5AC* via antisense morpholino resulted in shorter cupulae in zebrafish lateral line. Additionally, we generated zebrafish *muc5AC* mutants using CRISPR/Cas9 and found that cupulae in *muc5AC* mutants were significantly shorter than that in wild-types, but the hair cell number in neuromasts was not changed obviously. Furthermore, *muc5AC* mutant zebrafish larvae displayed compromised sensitivity to vibration stimuli compared to wild-type larvae. This study provides the first evidence linking the *muc5AC* gene to cupula development and vibration detection in zebrafish. Our findings suggest that Mucin-5AC is likely a critical component of the cupula matrix, offering an important clue to the molecular composition of the lateral line cupula in fish.

## 1. Introduction

The mechanoreceptive lateral line system, found in fish and amphibians, is essential for detecting water movement, which is involved in behaviors such as prey detection, predator avoidance, shoaling, and rheotaxis [1,2,3]. This sensory system comprises two types of sensory organs: superficial neuromasts, which are located on the surface of the body, and canal neuromasts, which are embedded within a canal [4]. Both types of neuromasts are composed of mechanosensory hair cells surrounded by support cells. The hair cells are submerged into a transparent gelatinous cupula, which interacts directly with water flow and transmits mechanical stimuli to the hair cells [4,5,6,7]. The cupula is present in all lateral line systems of gnathostome fishes [6].

Since its discovery, the morphology, composition, and function of the cupula have been studied in numerous fish species [4]. The cupulae of the lateral line sensory organs, which cover the entire neuromast and extend across the kinocilium, are typically cube- or dome-shaped. The length and diameter of the cupula vary between species and developmental stages, with cupula length generally correlated with the sensitivity of mechanoreception in fish [7,8,9,10]. Despite their varying morphologies, cupulae are primarily composed of mucopolysaccharides [11,12], which give them a much lower Young’s modulus compared to the kinocilia of the hair cell [13]. It has been reported that these mucopolysaccharides are synthesized in the mantle and support cells of the neuromast [14,15,16,17]. However, the precise composition of the neuromast cupula matrix remains largely unknown.

Mucins, high-molecular-weight glycoproteins, are key components of mucus hydrogels that line the epithelia of the respiratory, gastrointestinal, and urogenital tracts. These glycoproteins are mainly classified into two types: secreted gel-forming mucins (e.g., MUC2, MUC5AC, MUC5B) or transmembrane mucins (e.g., MUC1, MUC3A, MUC3B) [18]. In addition to the large central glycosylated proline, threonine, and serine (PTS) domain, gel-forming mucins are characterized by N- and C-terminal von Willebrand type D (vWD), von Willebrand C (vWC), and cysteine knot (CK) domains [19]. MUC2 or MUC5 contain cysteine-rich domains (CysD), with a conserved WxxW motif that is present within the PTS domains. These CysD domains are disulfide-rich modules that mediate intermolecular and intramolecular adhesion during mucin polymer assembly and may also play a role post-secretion in extracellular hydrogels [20].

Several studies have suggested mucins play an important role in sensory organ physiology. For instance, the cupula of the semicircular canal in toadfish (*Opsanus tau*) contains high concentrations of mucins [21]. Additionally, a mucin-like keratan sulfate glycopolymer found in the ampullae of Lorenzini in elasmobranch fish aids in detecting weak electric fields emitted from prey [22]. Another mucin, Otogelin, is localized in the tectorial membrane of the inner ear and is essential for hearing [23,24]. However, it remains unclear whether mucins are components of the neuromast cupula.

In this study, we found that the gene *muc5AC*, which encodes a novel family of mucin proteins with varying numbers of CysD domains, was specifically expressed in support cells in zebrafish neuromasts. We generated a stable *muc5AC* mutant zebrafish line and demonstrated that the cupulae of mutants were significantly shorter than those of wild-type zebrafish. Moreover, the startle response to vibration stimuli was compromised in the mutants. These results provide the first evidence of a mucin specific to the lateral line cupula and suggest its involvement in avoidance behavior.

## 2. Results

### 2.1. Structure of Zebrafish Mucin-5AC

To investigate the protein composition of the lateral line cupula, we searched the ZFIN database (http://zfin.org/ accessed on 22 July 2023) and identified a protein-coding gene, *muc5AC* (XM_684482, previous name is *si:dkey-205h13.2*), which is highly and specifically expressed in neuromasts and the lateral line ganglion in zebrafish larvae. This gene encodes a putative protein, Mucin-5AC, which contains an N-terminal signal peptide (SP, residues 1–24), and six CysD domains with a conserved WxxW motif, homologous to the DysD domains of human MUC2 and MUC5. The absence of transmembrane domains or subcellular localization signals suggests that Mucin-5AC is a secreted protein. A BLASTP analysis identified homologous proteins across species, including cartilaginous and bony fish, with sequence identities ranging from 42.23% to 75.31% compared to zebrafish Mucin-5AC (Table 1).

**Figure 1:**
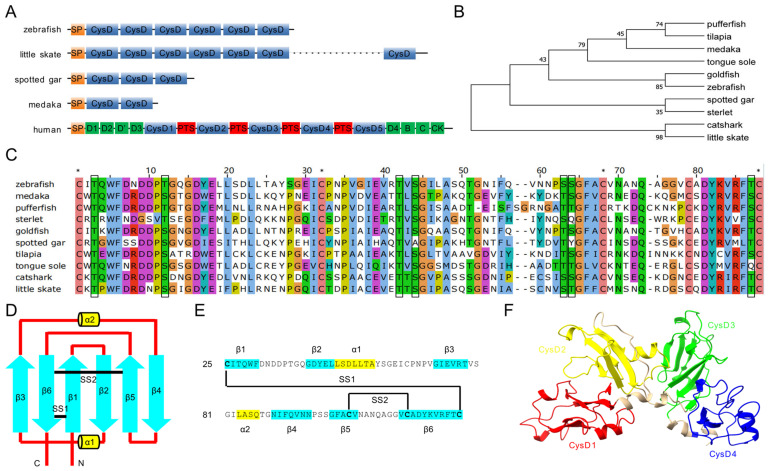
Structure, multiple alignment, and phylogenetic tree of Mucin-5AC proteins. (**A**) Schematic diagram of Mucin-5AC domains in zebrafish, little skate, spotted gar, medaka, and human. SP, signal peptide; CysD, cysteine rich domain; PTS, central proline, threonine, serine (PTS)-rich sequence. Green bars represent von Willebrand domains. (**B**) Phylogenetic tree of the first or second CysD domains of Mucin-5ACs. (**C**) Multiple alignment of the first or second CysD domain of Mucin-5ACs. Asterisks represent conserved cysteines (C). Black boxes indicate conserved serines (S) and threonines (T). (**D**) Secondary structure element of CysD1 in zebrafish Mucin-5AC. (**E**) Amino-acid sequence arrangement of CysD1 in zebrafish Mucin-5AC. The blue arrows (β1–β6) represent anti-parallel beta-strands and the yellow cylinders (α1–α2) represent short alpha helices. The black lines (SS1–SS2) mark disulfide bonds. (**F**) Three-dimensional structure of zebrafish Mucin-5AC (CysD1-CysD4) predicted by Alphafold.

Despite all the fish Mucin-5AC homologs containing CysD domains, the number of CysD domains vary across species, with, for instance, two CysD domains in Japanese medaka (*Oryzias latipes*), three in sterlet (*Acipenser ruthenus*), and twenty-seven in little skate (*Amblyraja radiata*). Unlike human Mucin-5AC, which contains vWFD and vWFC domains at its N- and C-terminal regions and PTS domains between the CysD domains, fish Mucin-5AC is structurally distinct (Figure 1A). The phylogenetic tree of the first or second CysD domains in these fishes reflects species relationships (Figure 1B). Multiple sequence alignment of these CysD domains shows five conserved cysteines involved in disulfide bond formation and eight conserved serine/threonine residues for O-linked glycosylation in fish Mucin-5AC (Figure 1C). Alphafold-predicted 3D structures reveal that zebrafish Mucin-5AC forms a globular tertiary structure with six anti-parallel beta sheets and two alpha helices. The CysD domains form a continuous chain, suggesting that Mucin-5AC may play a role in forming a specialized hydrogel in fish (Figure 1D–F).

### 2.2. muc5AC Is Exclusively Expressed in Neuromasts in Zebrafish

**Figure 2:**
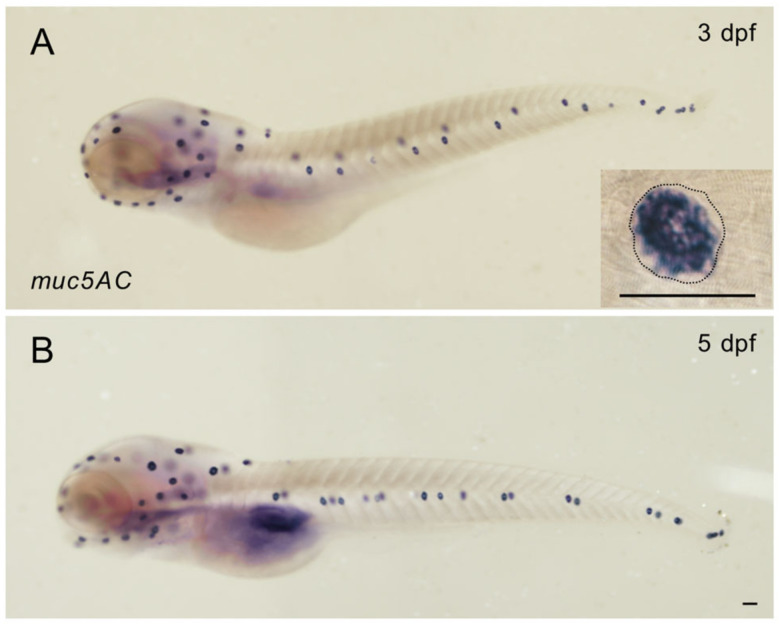
Zebrafish *muc5AC* is specifically expressed in neuromasts. (**A**) Representative image of whole-mount in situ hybridization of *muc5AC* at 3 dpf. Inset is the enlarged image of a neuromast in (**A**). Dashed line shows the outline of the neuromast. (**B**) Representative image of whole-mount in situ hybridization of *muc5AC* at 5 dpf. Scale bar of the inset, 50 μm; Scale bar of (**A**,**B**), 500 μm.

To illustrate the localization of fish Mucin-5AC, we performed whole-mount in situ hybridization in zebrafish larvae. Our results showed that *muc5AC* is strongly expressed in the neuromasts in zebrafish larvae at 3 days post-fertilization (dpf) and 5 dpf, which is consistent with previous findings about *si:dkey-205h13.2* gene [25,26,27]. Interestingly, the expression level of *muc5AC* was weaker in the center of the neuromast compared to the surrounding regions. *muc5AC* is absent in interneuromast cells, and its expression is confined to a smaller region than the entire neuromast (Figure 2A,B). In addition, we did not detect any *muc5AC* expression in the lateral line ganglion at 5 dpf, contrary to the ZFIN database description. This discrepancy suggests that the previous description of *si:dkey-205h13.2* expression in the ganglion may be incorrect.

### 2.3. muc5AC Is Specifically Expressed in the Support Cells in Neuromast

**Figure 3:**
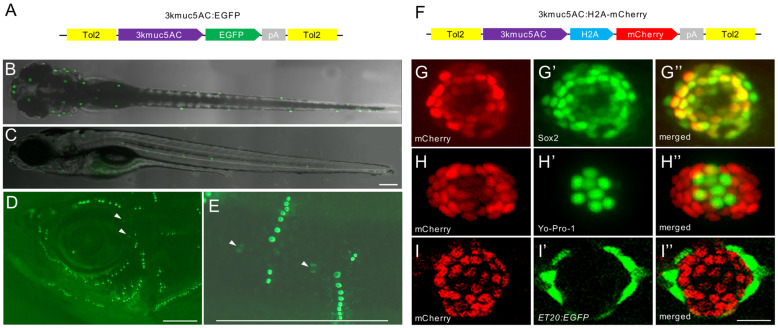
Characterization of the expression pattern of *Tg(3kmuc5AC:EGFP)* and *Tg(3kmuc5AC:EGFP)*. (**A**) Schematic diagram of the Tol2 construct used to generate the *Tg(3kmuc5AC:EGFP)* reporter. (**B**,**C**) Dorsal view and lateral view of a *Tg(3kmuc5AC:EGFP)* larva at 5 dpf. Scale bars: 200 μm. (**D**,**E**) Head and posterior region of operculum shown by *Tg(3kmuc5AC:EGFP)* in adult zebrafish. White arrows label canal neuromasts. Scale bars in D and E: 1 mm. (**F**) Schematic diagram of the Tol2 construct used to generate the *Tg(3kmuc5AC:H2A-mCherry)* reporter. (**G**–**G”**) Immunostaining with anti-mCherry (red) and anti-Sox2 (green). (**H**–**H”**) Live imaging of *Tg(3kmuc5AC:H2A-mCherry)* (red) stained with Yo-Pro-1 (green). (**I**–**I”**) Live imaging of *Tg(3kmuc5AC:H2A-mCherry)* (red) and *Tg(ET20:EGFP)* (green) double transgenic line. Scale bars: 20 μm.

To visualize the expression of *muc5AC* in vivo, we generated stable transgenic zebrafish lines using the 3 kb *muc5AC* promoter (Figure 3A). In the *Tg(3kmuc5AC:EGFP)* line, EGFP was specifically expressed in neuromasts, but not in interneuromast cells and the lateral line ganglion at 5 dpf, confirming that this transgenic line recapitulates endogenous *muc5AC* expression (Figure 3B,C). EGFP expression was also observed in both superficial and canal neuromasts in adult fish (Figure 3D,E), suggesting that Mucin-5AC is a constitutive component of neuromasts.

To investigate the specific cell types expressing *muc5AC*, we generated the *Tg(3kmuc5AC:H2A-mCherry)* line, which marks the nuclei of *muc5AC*-expressing cells (Figure 3F). We used Yo-Pro-1 staining for hair cells, Sox2 immunostaining for support cells, and the *Tg(ET20:EGFP)* line for mantle cells. The results revealed that mCherry expression was co-localized with Sox2-positive support cells but distinct from hair cells and mantle cells (Figure 3G–I”), indicating that *muc5AC* is specifically expressed in support cells of the neuromast. The Mucin-5AC protein, tagged with EGFP under the control of the 3 kb *muc5AC* promoter, also exhibited detectable expression in neuromasts in F0 larvae (Supplementary Figure S1).

### 2.4. Deficiency of muc5AC Reduces Cupula Length

**Figure 4:**
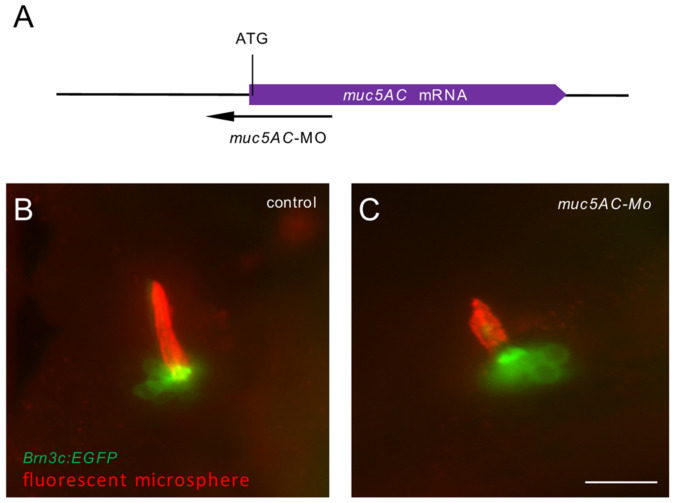
Knockdown of *muc5AC* reduces the length of cupulae in zebrafish larvae. (**A**) Schematic diagram of the target of *muc5AC*-Mo in *muc5AC* mRNA. (**B**,**C**) Live imaging of the larvae injected with control-Mo (**B**) and *muc5AC*-Mo (**C**) at 5 dpf. The hair cells and cupula are shown by *Tg(Brn3c:EGFP)* (green) and fluorescent microsphere (red), respectively. Scale bars: 50 μm.

**Figure 5:**
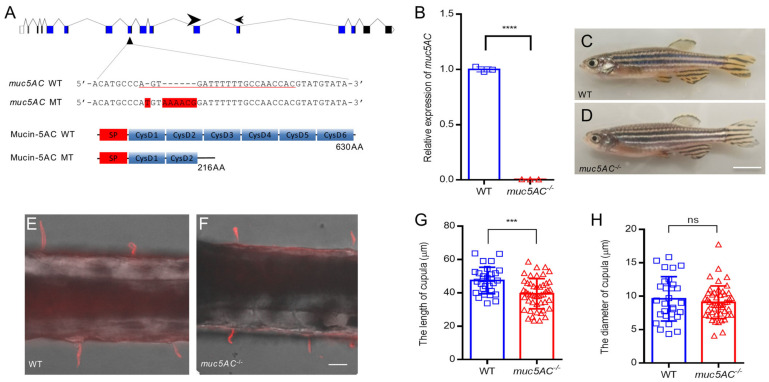
Mutation of *muc5AC* reduces the length of cupula in zebrafish larvae. (**A**) Schematic diagrams of *muc5AC* gene knockout. Top panel shows the scheme of *muc5AC* gene locus. Blue bars represent coding exon. Black bars represent 3′-or 5′-UTR. Arrowhead marks the location of gRNA. Arrows show the position of primers for qRT-PCR. Middle panel shows the sequence around gRNA target in wild-types and mutants. The gRNA target sequence is underlined. The insertion nucleotides are marked with red background. Bottom panel shows the truncated Mucin-5AC protein predicted according to the DNA insertion. (**B**) qRT-PCR analysis of *muc5AC* gene in *muc5AC* mutant and wild-type larvae at 5 dpf. (**C**,**D**) Representative images of wild-type and *muc5AC* mutant zebrafish at 3 months post-fertilization (mpf). Scale bar: 5 mm. (**E**,**F**) Representative images of cupulae in wild-type and mutant larvae at 5 dpf. Scale bar: 50 μm. (**G**) Quantification of cupula length in wild-type and *muc5AC* mutant larvae at 5 dpf. (**H**) Quantification of cupula diameter in wild-type and *muc5AC* mutant larvae at 5 dpf. ***, *p* < 0.001; ****, *p* < 0.0001; ns, not significant.

To gain insight into whether *muc5AC* plays a role in zebrafish cupula development, we used antisense morpholino oligonucleotides (MO) to knock down *muc5AC* expression (Figure 4A). The zebrafish embryos injected with *muc5AC*-MO displayed no obvious morphological changes. However, using *Tg(Brn3c:GFP)* zebrafish to label the hair cells in neuromasts, we observed through fluorescent microsphere staining that the cupula length in *muc5AC*-MO-injected larvae was shorter than that in control larvae at 5 dpf (Figure 4B,C). These data suggest that *muc5AC* is involved in cupula growth. We further validated these findings by generating a stable *muc5AC* mutant zebrafish line using Clustered Regularly Interspaced Short Palindromic Repeats (CRISPR)/ CRISPR-associated protein 9 (Cas9). A 7 bp insertion in exon 8 resulted in a premature stop codon, producing a truncated protein with only one complete CysD domain (Figure 5A). Compared to wild-type siblings, *muc5AC* mutants showed significantly reduced *muc5AC* mRNA levels, indicating nonsense-mediated decay (Figure 5B). Homozygous mutants are viable and fertile, with no visible morphological defects (Figure 5C,D). However, the cupulae of *muc5AC* mutants were significantly shorter than those of wild-type siblings at 5 dpf (wild-type: 47.4 ± 8.0 μm; *muc5AC*^−/−^: 38.2 ± 7.9 μm, *p* < 0.001) (Figure 5E–G), while the diameter of the cupula was not significantly changed (wild-type: 9.7 ± 3.3 μm; *muc5AC*^−/−^: 9.1 ± 2.6 μm, *p* = 0.43) (Figure 5E,F,H). Hair cell numbers were also not significantly changed in the mutants (Supplementary Figure S2). These results indicated that *muc5AC* was required for cupula growth in zebrafish neuromasts.

### 2.5. The Startle Response to Vibration Stimuli Is Compromised in muc5AC Mutants

**Figure 6:**
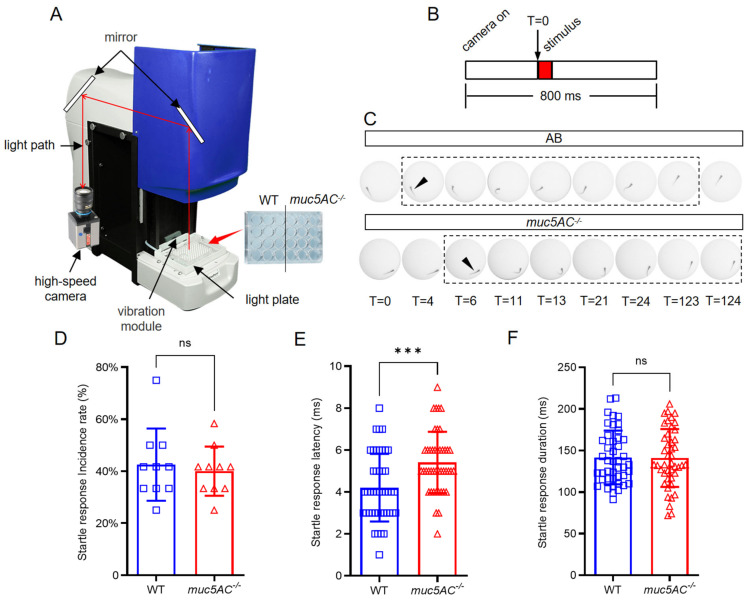
Deficiency of *muc5AC* increases the startle response latency. (**A**) Schematic diagram of the setup for vibration-induced startle response assay. The vibration module in Zebrabox is set to 70% maximum intensity and a frequency of 400 Hz. A high-speed camera is used to detect the movement of larvae. (**B**) Schematic diagram of the startle response experiment. T = 0 is the time when vibration stimuli is delivered. (**C**) Stages of the startle response of wild-type and *muc5AC* mutant larvae at 5 dpf. T = 0 represents stimulus onset. Arrowheads point to the detectable movement. Dashed rectangles highlight the whole process of startle response. T, ms. (**D**) Quantification of the short-latency startle response incidence rate in wild-type and *muc5AC* mutant larvae at 5 dpf. (**E**) Quantification of the short-latency startle response time in wild-type and *muc5AC* mutant larvae at 5 dpf. (**F**) Quantification of the startle response duration time in wild-type and *muc5AC* mutant larvae at 5 dpf. ***, *p* < 0.001; ns, not significant.

The lateral line is essential for detecting vibration stimuli and triggering startle responses in fish [28,29]. We assessed the escape response of *muc5AC*^−/−^ mutants to vibrational stimuli using Zebrabox (ViewPoint, Lyon, France) (Figure 6A,B). Both wild-type and *muc5AC*^−/−^ mutant larvae at 5 dpf exhibited a stereotyped C-start startle response upon encountering a sudden stimulus (Figure 6C) as described previously [30,31]. The occurrence rate of startle response was comparable between wild-type and *muc5AC* larvae (Figure 6D). However, the short-latency startle response occurred significantly later in *muc5AC* mutants than in wild-type larvae (wild-type: 4.2 ± 1.6 ms; *muc5AC^−/−^*: 5.4 ± 1.5 ms, *p* < 0.001) (Figure 6E), indicating a slower response to potential threats in mutants. Despite this delay, the overall duration of the startle response remained similar between the two groups (wild-type: 141.7 ± 32.2 ms; *muc5AC^−/−^*: 141.0 ± 34.8 ms, *p* = 0.92) (Figure 6F), suggesting that the mutants’ locomotion capacity was not impaired. These findings indicate that the *muc5AC* gene is required for a rapid startle response in zebrafish larvae.

## 3. Discussion

In this study, we identified a novel family of mucin, Mucin-5AC, which is conserved across most cartilaginous and bony fishes. Our findings demonstrate that *muc5AC* is specifically expressed in the support cells of zebrafish neuromasts. The mutation of *muc5AC* results in shorter cupulae in neuromasts and delays the startle response to vibrational stimuli, suggesting that Mucin-5AC is a key component of the cupula. These insights enhance our understanding of the development and function of the lateral line system in fish.

The cupula, which serves as the interface between hair cells and the surrounding water, is crucial for transmitting water flow stimulus to hair cells [32]. Its matrix composition directly influences its mechanical properties, which are essential for sensory detection. Previous studies have shown that the cupula is primarily composed of mucopolysaccharides, as evidenced by periodic acid–Schiff (PAS) staining [11,33]. Our results suggest that Mucin-5AC could be a major component of the neuromast cupula in zebrafish. Mucins have previously been identified in the ampullae of Lorenzini and the inner ear [22,23,24]. Given its high serine and threonine content, Mucin-5AC likely organizes the mucopolysaccharides in the neuromast cupula. Furthermore, its conservation across cartilaginous and bony fishes, which all possess cupulae, suggests that Mucin-5AC plays a critical role in cupula formation. The exclusive expression of *muc5AC* in neuromasts and the reduction in cupula length in *muc5AC* mutants further support its role in cupula growth. As our study focused exclusively on zebrafish, the role of Mucin-5AC in the lateral line cupula needs investigation across a broader range of fish species. For species possessing an ortholog of zebrafish *muc5AC*, it is essential to examine whether *muc5AC* is expressed in the neuromasts. Additionally, the morphology of the cupula in species lacking a *muc5AC* ortholog should be compared with that of species that possess it. Such comparative analyses could provide valuable insights into the functional and structural significance of Mucin-5AC in the lateral line system across different fish taxa.

**Figure 7:**
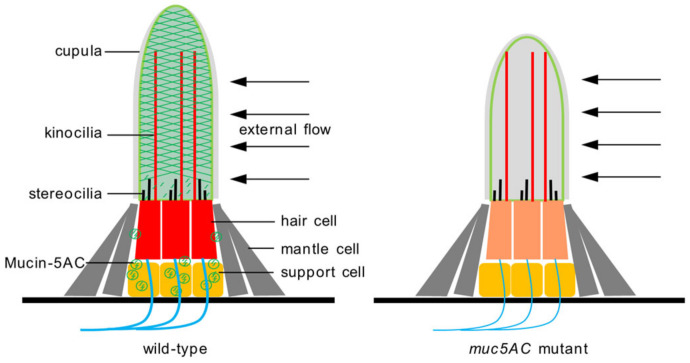
Schematic diagram of the cupula in wild-type and *muc5AC* mutant neuromasts. Mucin-5AC is synthesized in support cells and assembled into polymer hydrogels around the cilium bundle.

The cupula is composed of two layers: a central layer above the support cells, and an outer layer above the mantle cells [6,15]. Both support and mantle cells are believed to synthesize the glycoproteins that form these layers [14,33]. Our results indicate that *muc5AC* encoding a secretory protein is specifically expressed in support cells and subsequently secreted from these cells, suggesting its potential role in the central layer of the cupula. The shorter cupulae observed in *muc5AC* mutants are likely due to defects in the central layer, while the outer layer remains structurally intact (Figure 7). The upregulation of *muc5AC* at 24 h post-treatment (hpt), when many hair cells begin to regenerate, further supports its involvement in cupula formation (Supplementary Figure S3). To investigate the secretion and assembly process of Mucin-5AC in the neuromast, we constructed plasmids encoding Mucin-5AC with EGFP tags at both the N- and C-termini. In F0 larvae injected with the N-terminal construct, fluorescence was specifically observed in the neuromast but not in the cupula. However, the chimeric transgene in F0 larvae typically produces insufficient levels of the transgenic protein. Establishing a stable transgenic line would facilitate a clearer understanding of the localization of Mucin-5AC within the neuromast. Alternatively, generating antibodies against Mucin-5AC and performing immunofluorescence assays could provide an effective solution for visualizing its precise localization. Although we were unable to directly visualize Mucin-5AC proteins in the cupula, our findings strongly suggest its critical role in cupula development.

The lateral line system is essential for various behaviors in fish, including prey detection, predator avoidance, shoaling, and rheotaxis. In this study, *muc5AC* mutant larvae, which had shorter cupulae in their neuromasts, exhibited delayed responses to vibrational stimuli compared to wild-type zebrafish. Consistent with our findings, McHenry et al. [34] suggested that cupula height is a critical morphological factor for frequency response sensitivity. Furthermore, cavefish, which exhibit increased sensitivity to low-frequency vibrations, possess larger cupulae compared to surface fish [8,9]. Given the influences of *muc5AC* on cupula length in zebrafish, it would be valuable to investigate whether differences in *muc5AC* expression level exist between cavefish and surface fish.

The zebrafish neuromast serves as a valuable model for studying hair cell regeneration, as it contains heterogeneous cell populations, including hair cells, support cells, and mantle cells [17,35,36]. Various transgenic zebrafish lines have been developed for lineage tracing and studying hair cell regeneration. For instance, the *myo6b* promoter can drive the expression of the human diphtheria toxin receptor (hDTR) in hair cells, enabling specific ablation [37]. Other regulatory elements, such as the *She* enhancer for support and mantle cells [38] and the *alpl* enhancer for mantle cells [39], allow precise manipulation of these cell types. While support cells are crucial for hair cell regeneration, there are currently few transgenic lines that specifically target support cells. In this study, we demonstrated that the 3 kb *muc5AC* regulatory sequence can specifically drive the expression of EGFP and H2A-mCherry in support cells. These transgenic lines could be used to profile the support cell transcriptome and investigate their role in hair cell regeneration. Moreover, by using a transgenic line expressing EGFP-tagged ribosomal proteins under the control of the *muc5AC* promoter, we can analyze the translating mRNA profiles of support during hair cell regeneration using translating ribosomal affinity purification (TRAP) [40].

## 4. Materials and Methods

### 4.1. Zebrafish Maintenance

The zebrafish (*Danio rerio*) is a widely used model organism for studying vertebrate biology. Wild-type zebrafish (AB strain) and transgenic lines, including *Tg(Brn3c:GFP)* for hair cell labeling and *Tg(ET20:EGFP)* for mantle cell labeling, were maintained in a circulating system at 26–28 °C under a 14 h light/10 h dark cycle. Illumination was provided by four 20 W LED lamps. Each of the 10–15 adult zebrafish was maintained in 3 L plastic tank and fed artemia twice daily at 9:00 AM and 5:00 PM.

Embryos were obtained through natural spawning with one male and one female and reared in blue water (60 mg/L sea salt, 0.5 mg/L methylene blue) at a density of 50–100 embryos per dish. They were maintained at 28.5 °C in darkness until 5 dpf. Embryos and larvae were staged as described previously [41]. All experimental procedures were conducted in accordance with guidelines approved by the Ethics Committee of Shanghai Ocean University.

### 4.2. Sequence Alignment, Phylogenetics, and Structural Analysis

Homologous proteins of zebrafish Mucin-5AC were retrieved from the GenBank protein database using BLASTP (NCBI). The accession numbers of these homologous proteins are listed in Table 1. The first or second CysD domains of these proteins were aligned using the CLUSTALW method. Phylogenetic analysis was performed using the neighbor-joining method using 1000 bootstrap replicates in MEGA X. The 3D structures of zebrafish Mucin-5AC were predicted using the AlphaFold database [42].

### 4.3. Whole-Mount In Situ Hybridization

Zebrafish larvae were fixed in 4% paraformaldehyde (PFA) at 4 °C overnight and then dehydrated in a series of methanol gradients. Probe generation and hybridization were performed as described previously [43]. A DNA fragment of zebrafish *muc5AC* was amplified from embryonic cDNA using primers listed in Supplementary Table S1. RNA probes were transcribed from cDNA using T7 RNA polymerase (Vazyme, Nanjing, China) and DIG RNA Labeling Mix (Roche, Basel, Switzerland). Hybridization was carried out at 65 °C for 16 h. Alkaline phosphatase-conjugated digoxigenin antibodies (1:2000; Roche, Basel, Switzerland) were used for detection, and BM purple was used for visualization (Roche, Basel, Switzerland). Samples were mounted in glycerol and imaged on a Nikon Eclipse 80i microscope (Nikon, Tokyo, Japan).

### 4.4. Transgenic Zebrafish Lines Generation

The 3 kb promoter region upstream of the zebrafish *muc5AC* transcription start site (chr23:26535840-26539034) was amplified from the zebrafish embryonic genomic DNA. The p3kmuc5AC:EGFP construct was created by replacing the NF-κB recognition sequences in pNFkB:EGFP (addgene:44922) [44] with the 3 kb *muc5AC* promoter (Figure 3A). To generate the p3kmuc5AC construct, the EGFP in p3kmuc5AC was replaced with H2A-mCherry from p3E-mCherrypA (Tol2Kit) via seamless cloning (Vazyme, Nanjing, China) (Figure 3F). The recombinant plasmids were validated by sequencing. Transgenic zebrafish lines were generated by Tol2-mediated transposition [45]. Approximately 25 ng plasmids and 50 ng Tol2 transposase mRNA were injected into one-cell stage embryos. The injected embryos were raised in blue water and screened for fluorescence at 3 dpf. F2 progeny with stable inheritance were used for the experiments.

### 4.5. Gene Knockdown with Antisense Morpholino

A morpholino antisense oligonucleotide (Gene Tools, Philomath, OR, USA) targeting the translation start site of zebrafish *muc5AC* was designed (5′-TGGTGTTCATTTTTCTGGCTTTTCT-3′). A standard nonspecific morpholino was used as a control. Each one-cell stage zebrafish embryo was injected with 50 ng *muc5AC*-MO according to common practice [46], and the injected embryos were raised in blue water. Phenotype observations and overall gross morphology were visualized using an inverted microscope at 5 dpf.

### 4.6. Gene Knockout Using CRISPR/Cas9

Zebrafish *muc5AC* mutants were generated by CRISPR/Cas9 as described by Varshney et al. (2017) [47]. Guide RNAs targeting *muc5AC* (XP_689574) were designed using CRISPRScan (oligo sequences in Supplementary Table S1). The sgRNAs and capped NLS-Cas9 mRNA were transcribed using the T7 High Yield RNA Transcription Kit (Vazyme, Nanjing, China) and mMessage mMachine T7 Transcription Kit (Thermo Fisher, Waltham, MA, USA). Approximately 1.4 nL of mixture containing 300 ng Cas9 mRNA and 50 ng sgRNA was injected into the one-cell stage embryos. The efficiency of the gRNA was evaluated by fluorescent PCR followed by capillary electrophoresis [48], using the primers listed in Supplementary Table S1. Progeny were raised to adulthood and sequenced to confirm mutations.

### 4.7. Quantitative RT-PCR (qRT-PCR)

qRT-PCR was performed as previously described [49]. Total RNA was extracted from 10–20 embryos at 5 dpf using TRIzol reagent (Invitrogen, Waltham, MA, USA). cDNA was generated by the HiScript III 1st Strand cDNA Synthesis Kit (Vazyme, Nanjing, China) using random primers and qPCR was performed with the Hieff UNICON Universal Blue qPCR SYBR Green Master Mix (YEASEN, Shanghai, China) on an ABI PRISM 7000 Real-Time PCR System (Applied Biosystems, Waltham, MA, USA). The eef1a1l1 gene served as the endogenous control. The primers are listed in Supplementary Table S1. All reactions were performed in triplicate, and the relative gene expression of each gene was calculated using the 2-∆∆Ct method and normalized to control group levels.

### 4.8. Hair Cell Staining with YO-PRO-1

YO-PRO-1 (C24H29I2N3O, Molecular Probes, Eugene, OR, USA) is a cyanine monomer dye that can enter into the hair cell through large nonselective channels and bind to DNA. Hair cells in the zebrafish neuromast were stained with the vital dye YO-PRO-1 as described previously [50,51]. The larvae were incubated with 2 μM YO-PRO-1 for 1 h at 28.5 °C in the dark. After washing with Holtfreter’s buffer (3.5 g/L NaCl, 0.05 g/L KCl, 0.1 g/L CaCl_2_, 0.025 g/L NaHCO_3_, and 5 mM HEPES (pH 7.0)), the larvae were anesthetized with 0.01% tricaine methanesulfonate (MS-222, Sigma, St. Louis, MO, USA), placed in a glass bottom dish, and visualized under an inverted fluorescence microscope (Zeiss Axio Observer Z1, Oberkochen, Germany). The first and second neuromasts in the posterior lateral line were selected for hair cell quantification.

### 4.9. Cupula Staining with Fluorescent Microspheres

To visualize the cupula in zebrafish neuromasts, fluorescent microspheres (100 nm, Zhichuan, Shanghai, China) were used as described by Yoshizawa et al. [9]. The larvae were briefly anesthetized on ice and then exposed to microsphere solution (diluted 1:5 in Holtfreter’s buffer) for 1–2 min. After rinsing, samples were embedded in 2% methylcellulose and visualized using a DsRed filter on a fluorescence microscope (Zeiss, Oberkochen, Germany). The first and second neuromasts were used for quantification.

### 4.10. Immunofluorescence

Larvae were fixed in 4% PFA at 4 °C overnight, dehydrated with methanol, and stored at −20 °C. After washing with PBDT (PBS/1% DMSO/0.1% Tween-20), samples were blocked with 2.5% normal goat serum (Invitrogen, Waltham, MA, USA) for 2 h at room temperature. Monoclonal mouse anti-mCherry (1:200, ABclonal, Wuhan, China) and rabbit anti-Sox2 (1:200, Sigma, St. Louis, MO, USA) were also used as primary antibodies and diluted in 2.5% normal goat serum (Invitrogen, Waltham, MA, USA). After three rinses with PBDT, samples were incubated in goat anti-rabbit Alexa Fluor 488 conjugated IgG (1:200, Invitrogen, Waltham, MA, USA) and goat anti-mouse Alexa Fluor 594 conjugated IgG (1:200, Invitrogen, Waltham, MA, USA) for 2 h at room temperature. Samples were imaged using an Axio Observer Z1 microscope (Zeiss, Oberkochen, Germany) equipped with a Plan-Apochromat 20x/0.8 M27 objective lens (Zeiss, Oberkochen, Germany).

### 4.11. Vibration-Induced Startle Response Assay

The vibration-induced startle response assay was conducted using zebrafish larvae at 5 days post-fertilization (dpf) at room temperature. Twelve wild-type and twelve *muc5AC* mutant larvae were individually placed into the wells of a 24-well plate filled with Holtfreter’s buffer (one larva per well). The plate was then positioned inside a Zebrabox system (ViewPoint, Lyon, France) with a light intensity of 320 lux. As previously reported, startle responses can be triggered by vibration stimuli within a frequency range of 50–1000 Hz [30,31]. Following a 10 min acclimation period, the Zebrabox acoustic generating system was used to deliver a 5 millisecond vibration stimulus at 70% maximum intensity and a frequency of 400 Hz, which elicited the most pronounced startle response. The response was recorded using a high-speed camera (J-PRI, AOS, New York, NY, USA) at 1000 frames per second for a duration of 800 milliseconds. A total of 10 trials were conducted. Three parameters were recorded for analysis: startle response frequency, startle latency, and response duration. Startle response frequency was calculated as the percentage of larvae exhibiting a startle response (i.e., startle response incidence rate). Startle latency was defined as the time from the onset of the stimulus to the first detectable head movement. Response duration was measured as the time from the initial head movement to a state of relative calmness, indicated by the absence of bending in the subsequent five frames.

### 4.12. Data Statistical Analysis

All statistical analyses were carried out using GraphPad Prism 8.0. For comparison between two groups, an unpaired *t*-test with equal variance was employed. All data were displayed as mean with standard deviation (SD). *p* < 0.05 was regarded as statistically significant.

## 5. Conclusions

In this study, we identified Mucin-5AC as a novel family of mucins conserved in cartilaginous and bony fishes. *muc5AC* is specifically expressed in the support cells of zebrafish neuromasts, suggesting its critical role in the central layer of the cupula. Deficiency of *muc5AC* resulted in shorter cupulae in neuromasts and delayed startle responses to mechanical stimuli, suggesting that Mucin-5AC is the key component of the lateral line cupula in fish.

## Figures and Tables

**Table 1:** Mucin-5ACs in bony and cartilaginous fishes.

Common Name	Species Name	Accession No.	CysD No.	Identity to Zebrafish
little skate	*Amblyraja radiata*	XP_032903886	27	48.75%
smaller spotted catshark	*Scyliorhinus canicula*	XP_038629501	19	47.45%
sterlet	*Acipenser ruthenus*	XP_058858610	4	50.38%
spotted gar	*Lepisosteus oculatus*	XP_015192309	3	42.53%
goldfish	*Carassius auratus*	XP_026092345	6	75.31%
tongue sole	*Cynoglossus semilaevis*	XP_008309417	7	42.23%
Japanese medaka	*Oryzias latipes*	XP_004085768	2	47.89%
Nile tilapia	*Oreochromis niloticus*	XP_025754833	2	42.86%
pufferfish	*Takifugu rubripes*	XP_029692542	2	47.49%

## Data Availability

Data will be provided upon request.

## Supplementary Materials

The following supporting information can be downloaded at: https://www.mdpi.com/article/10.3390/ijms26020708/s1.
