# Supplementary material for: A Lateral Line Specific Mucin Involved in Cupula Growth and Vibration Detection in Zebrafish
Source: Int J Mol Sci. 2025 Jan 15;26(2):708. doi: 10.3390/ijms26020708 (PMC11765562; doi:10.3390/ijms26020708)
Supplement: Supplementary file 1 [file ijms-26-00708-s001.zip › ijms-3334983-supplementary.pdf]

Supplementary data

Table S1 Primer list.

Gene	Sequence (5'-3')	Application
muc5AC	TAATACGACTCACTATAGGGGTTGGCAAAAAATCACTGTTTTAGAGCTAGAA	gRNA
muc5AC	AAAAGCACCGACTCGGTGCCACTTTTTCAAGTTGATAACGGACTAGCCTTAT<br>TTTAACTTGCTATTTCTAGCTCTAAAAC	gRNA
muc5AC	F: GTGTCTTATGTTTCAAGAGCACAAGCGT<br>R: TGTAAAACGACGGCCAGTCAGAATCAAACCACCTGGTAAC	genotyping
muc5AC	F: CGTGGTTGGCAAAAAATCACTGG<br>F: CGTGGTTGGCAAAAAATCCGTT<br>R: TTCTCTCAGAGCATGGGGTG	genotyping
muc5AC	F: ACAGTCTCCGGCATACTAGC<br>R: TAATACGACTCACTATAGGAGGCCCCATTCACACACTTA	Probe
eef1a1l1	F: CTTCTCAGGCTGACTGTGC<br>R: CCGCTAGCATTACCCTCC	qRT-PCR
muc5AC	F: GAGGTCACAACCCTTAGCGG<br>R: ACCTGGTTACACACTGTCCA	qRT-PCR
3kmuc5AC	F: GGCCTCGAGGGTAAGACCGAGCCATCATGT<br>R: GGCGGATCCTGACCACCACTAAATGCAGGA	construction
mCherry	F: CAGGGGGGGTGAGCAAGGGCGAGGAG<br>R: TTACTTGTACAGCTCGTCCATG	construction
vector backbone	F: GACGAGCTGTACAAGTAAAGCG<br>R: TGCCATGGTGGCGGATCCTGACCAC	construction
H2A	F: ATCCGCCACCATGGCAGGTGGAAAAGCAGG<br>R: TTGCTCACCCCCCCTGCGGTTTTCT	construction

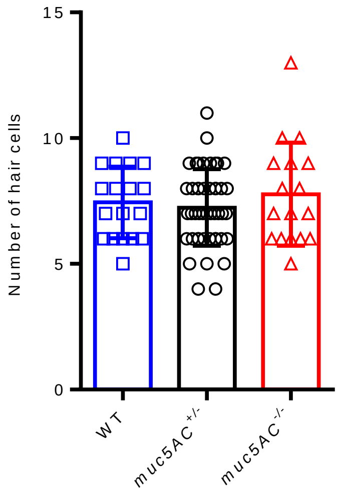

Figure S1 Quantification of hair cells in zebrafish posterior lateral line neuromasts using Yo-Pro-1 staining at 5 dpf.

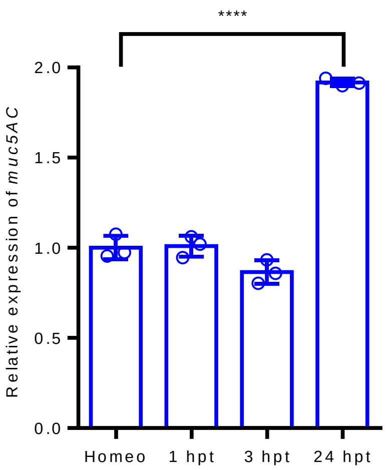

Figure S2 qRT-PCR analysis of *muc5AC* expression at 5 dpf (Homeo) and after neomycin treatment (1 hpt, 3 hpt, and 24 hpt). ****, p<0.0001.
